# Supplementary material for: Complex‐centric proteome profiling by SEC‐SWATH‐MS
Source: Mol Syst Biol. 2019 Jan 14;15(1):e8438. doi: 10.15252/msb.20188438 (PMC6346213; doi:10.15252/msb.20188438)
Supplement: Supplementary file 8 — Dataset EV7 [file MSB-15-e8438-s008.zip › feature_plots_string/Q969S9.pdf]

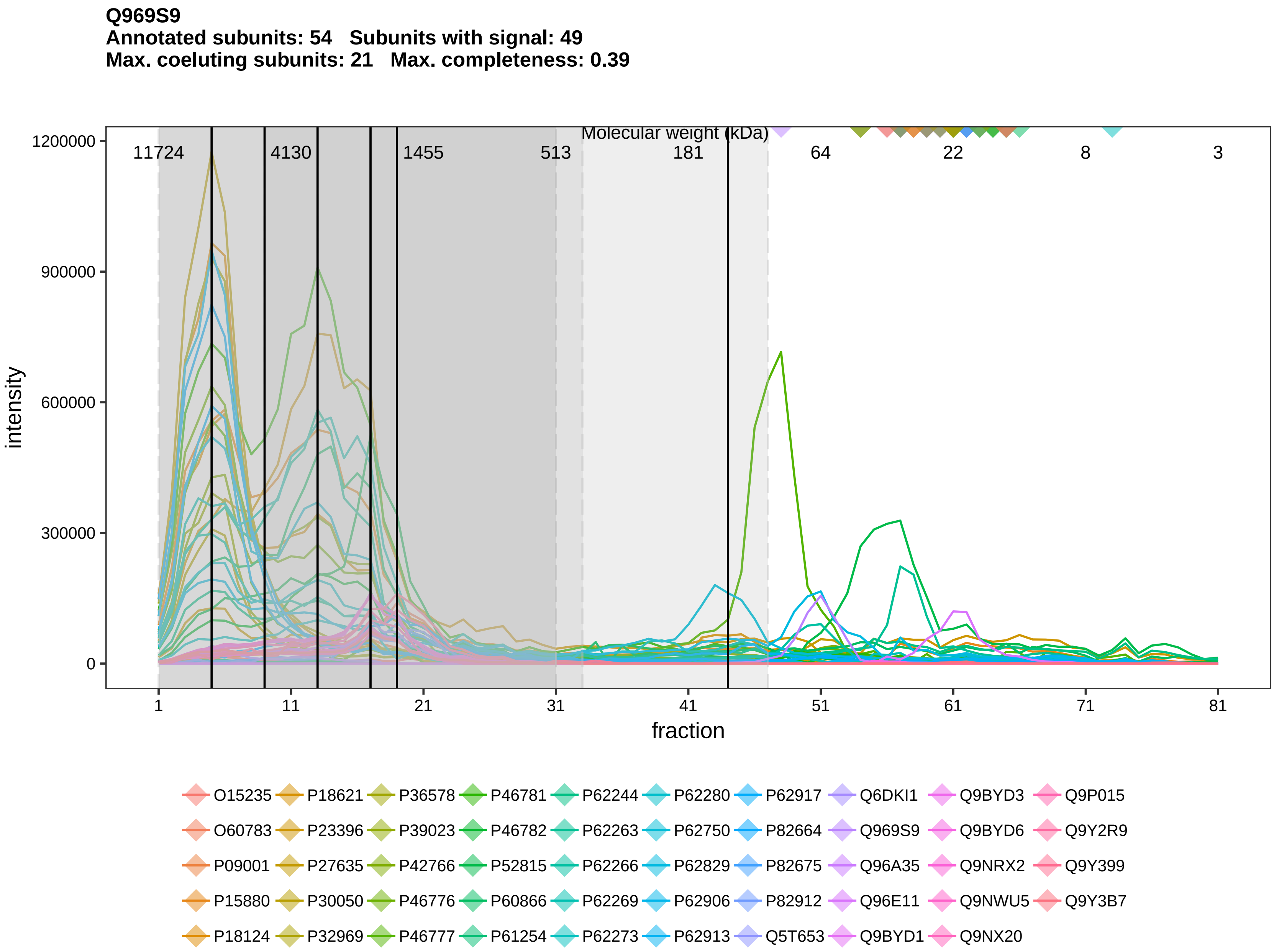

Q969S9
Annotated subunits: 54 Subunits with signal: 49
Max. coeluting subunits: 21 Max. completeness: 0.39
Molecular weight (kDa)
11724
4130
1455
513
181
64
22
8
3
intensity
1200000
900000
600000
300000
0
fraction
1
11
21
31
41
51
61
71
81
O15235
O60783
P09001
P15880
P18124
P18621
P23396
P27635
P30050
P32969
P36578
P39023
P42766
P46776
P46777
P46781
P46782
P52815
P60866
P61254
P62244
P62263
P62266
P62269
P62273
P62280
P62750
P62829
P62906
P62913
P62917
P82664
P82675
P82912
Q5T653
Q6DKI1
Q969S9
Q96A35
Q96E11
Q9BYD1
Q9BYD3
Q9BYD6
Q9NRX2
Q9NWU5
Q9NX20
Q9P015
Q9Y2R9
Q9Y399
Q9Y3B7
